# Graphene Oxide/ZnS:Mn Nanocomposite Functionalized with Folic Acid as a Nontoxic and Effective Theranostic Platform for Breast Cancer Treatment

**DOI:** 10.3390/nano8070484

**Published:** 2018-06-30

**Authors:** Daysi Diaz-Diestra, Bibek Thapa, Dayra Badillo-Diaz, Juan Beltran-Huarac, Gerardo Morell, Brad R. Weiner

**Affiliations:** 1 Molecular Sciences Research Center, University of Puerto Rico, San Juan, PR 00926, USA; bibech.thapa@gmail.com (B.T.); gerardo.morell@upr.edu (G.M.); bradrweiner@gmail.com (B.R.W.); 2 Department of Chemistry, University of Puerto Rico, San Juan, PR 00925-2534, USA; 3 Department of Physics, University of Puerto Rico, San Juan, PR 00925-2537, USA; 4 Department of Biology, University of Puerto Rico, San Juan, PR 00925-2537, USA; dnbd1118@hotmail.com; 5 Department of Environmental Health, Center for Nanotechnology and Nanotoxicology, Harvard T. H. Chan School of Public Health, Boston, MA 02115, USA

**Keywords:** drug delivery, quantum dots, reduced graphene oxide, chemotherapy, theranostics

## Abstract

Nanoparticle-based cancer theranostic agents generally suffer of poor dispersability in biological media, re-agglomeration over time, and toxicity concerns. To address these challenges, we developed a nanocomposite consisting of chemically-reduced graphene oxide combined with manganese-doped zinc sulfide quantum dots and functionalized with folic acid (FA-rGO/ZnS:Mn). We studied the dispersion stability, Doxorubicin (DOX) loading and release efficiency, target specificity, internalization, and biocompatibility of FA-rGO/ZnS:Mn against folate-rich breast cancer cells, and compared to its uncoated counterpart (rGO/ZnS:Mn). The results indicate that DOX is adsorbed on the graphene surface via π–π stacking and hydrophobic interaction, with enhanced loading (~35%) and entrapment (~60%) efficiency that are associated to the chelation of DOX and surface Zn^2+^ ions. DOX release is favored under acidic conditions reaching a release of up to 95% after 70 h. Membrane integrity of the cells assessed by Lactate dehydrogenase (LDH) release indicate that the surface passivation caused by folic acid (FA) functionalization decreases the strong hydrophobic interaction between the cell membrane wall and the edges/corners of graphene flakes. Chemotherapeutic effect assays reveal that the cancer cell viability was reduced up to ~50% at 3 µg/mL of DOX-FA-rGO/ZnS:Mn exposure, which is more pronounced than those obtained for free DOX at the same doses. Moreover, DOX-rGO/ZnS:Mn did not show any signs of toxicity. An opposite trend was observed for cells that do not overexpress the folate receptors, indicating that FA functionalization endows rGO/ZnS:Mn with an effective ability to discriminate positive folate receptor cancerous cells, enhancing its drug loading/release efficiency as a compact drug delivery system (DDS). This study paves the way for the potential use of functionalized rGO/ZnS:Mn nanocomposite as a platform for targeted cancer treatment.

## 1. Introduction

According to the US National Cancer Institute, approximately 1.7 million people in the US will be diagnosed with cancer during this year [1]. Most of the current treatment options, such as chemotherapy, radiation therapy and immunotherapy, suffer of poor specificity and debilitating side effects [2]. To address these drawbacks, theranostic approaches based on dendrimers, micelles and proteins are being used for targeted diagnosis and therapy with an increase in the treatment efficiency [3,4,5,6]. Most recently, functional nanomaterials have shown great potential to load and deliver clinical chemotherapy drugs, such as doxorubicin (DOX) and cisplatin, into targeted tissues [7,8]. These drug delivery systems (DDSs) have been reported to exhibit higher selectivity, solubility, stability and quick body clearance when compared to conventional free-drug molecule based systems [8]. 

Among the different nanomaterial-based DDSs reported to date, those based on quantum dots (QDs) offer superior advantages due to the exotic features of QDs, such as unique tunable optical properties, high quantum yield, photo-stability and broad absorption with relatively-narrow and symmetric emission, and the fact that they can be simultaneously used for bio-imaging cancerous tissues and as photosensitizers in photodynamic therapy (PDT) [9,10,11]. Nevertheless, this technology is limited to the use of DDSs that contain acutely toxic metals (such as cadmium and indium), which might get released from the QDs structure into cellular compartments causing serious toxic effects [12,13,14]. To overcome these toxicity issues, Cd-free QDs (such as ZnS) have also been explored and reported that they exhibit improved stability and bio-compatibility [15,16,17]. To further improve the optical properties of ZnS QDs, transition metal doping strategies have been adopted, yielding species including Mn doped ZnS (ZnS:Mn) QDs. ZnS:Mn QDs exhibit an additional orange emission band (centered around 590 nm) that provides large-effective Stokes shifts, multiplicity of decay times, photoluminescence intermittency under continuous excitation, additional optical tunability, and, most importantly, enable clinicians to avoid the use of organic acid-stabilized Cd-based systems and substitute their optical contribution with Mn doping in the ZnS matrix (e.g., for bioimaging) [18,19]. Various reports have indicated their suitability as drug delivery carriers and nano-probes showing no detectable toxic effects at low Mn doping levels [20,21,22,23]. Our research group recently found that ligand-capped ZnS:Mn systems can generate quantum yields as high as 50% in water, rendering them suitable as PDT photosensitizers for cancer treatment [9].

Given the ultra-small size of ZnS:Mn QDs (usually down to 5 nm), they tend to easily aggregate in biological media, which in turn affects their specific tumor accumulation that is critical in passive DDSs [24,25]. One reported solution has been the immobilization and homogeneous dispersion of surface-complexed QDs onto the active surface of functional nanocomposites, such as graphene [26,27,28]. Graphene, being a 2- dimensional nanomaterial composed of π conjugated benzene rings with high mechanical flexibility and electrical conductivity, interacts non-covalently with biomolecules, chemical species and exogenous mediators due to its delocalized π electron cloud [29,30,31]. In particular, graphene oxide (GOx) exhibits a high planar surface with abundant functional groups (including carboxyl, carbonyl and hydroxyl groups), which enables it to be coupled with imaging probes and drugs. GOx has been employed as a DDSs carrier for drugs, proteins and genes with an efficient release under acidic conditions [32,33,34]. 

Sun et al. reported the physisorption of DOX onto GOx (via π–π and electrostatic interactions) at a basic pH, which was then released at an acidic pH upon the increase of hydrophilicity and solubility of DOX [35]. Such pH-dependent release mechanism can be used to facilitate the accurate delivery of therapeutic agents into cancerous tissues without affecting normal tissues. Hu et al. reported that QDs tagged to reduced GOx (rGO) serve as imaging agents and PDT photosensitizers [27]. Chen et al. further developed a traceable DDS based on QDs conjugated to graphene and functionalized with transferrin (Trf) in which DOX was loaded to target cancer cells [26]. This system was shown to be capable of differential uptake and imaging of cancer cells that express the Trf receptor. 

Most of these QDs/graphene based-DDSs have been synthesized via stepwise approaches through covalent and non-covalent linking to mitigate the toxicity of QDs and decrease their fluorescent quenching. These synthesis approaches are generally cumbersome, time-consuming, and cost-ineffective. Moreover, the stepwise approaches generate additional defects on the graphene surface, which negatively affect the drug loading efficiency, specificity, and safety of the QDs conjugates for cancer imaging, monitoring, and therapy [36].

In contrast to the previous stepwise synthesis approaches, we performed the straightforward concerted synthesis of a new functional nanocomposite consisting of chemically-reduced GOx decorated with ZnS:Mn (rGO/ZnS:Mn). To inhibit the toxic response of this nanocomposite, we have functionalized it with folic acid (FA) via carbodiimide-mediated covalent crosslinking. FA is a ligand widely used in the construction of targeted DDSs given the overexpression of folate receptors in the membrane of cancer cells (compared for instance to normal cells) [37]. Moreover, FA is nontoxic, inexpensive, and quite stable in biological media. Zhang et al. were the first to report FA covalent functionalization of a graphene based drug nanocarrier [38] Their pH-responsive DDS efficiently released model drugs (e.g., DOX and Camptothecin) in folate positive receptor MCF-7 cancer cells inducing acute cytotoxicity higher than those found in folate negative receptor A549 cells, which was ascribed to the target specificity of FA. Adding FA to carbon-based nanocarriers has been reported to increase their dispersion stability and to reduce significantly its toxicity. Castillo et al. [39] reported a non-covalent FA functionalization of carbon nanotubes, which not only increases their target specificity to folate over-expressed cells, but also inhibits aggregation and re-agglomeration. More recently, Park et al. [40] reported a similar finding where FA functionalization of rGO serves as stabilizer to avoid its aggregation in physiological media, and to effectively enable the loading of anticancer drugs. The system showed high specific targeting to cancer cells and excellent drug-release efficiency resulting in acute cytotoxicity in vitro [40].

Hereby, we report the effect of FA functionalization on the rGO/ZnS:Mn nanocomposite in terms of dispersion stability, biocompatibility and target specificity. DOX was selected as a model drug and loaded on the nanocomposite surface; the loading was monitored through π–π stacking and hydrophobic interactions. We also evaluate the efficiency of this DDS to release DOX at acidic conditions (similar to endosomic pH in cancer cells), and its capability to discriminate positive folate receptor cancer cells.

## 2. Experimental Section

### 2.1. Synthesis of GOx

GOx was synthesized following the improved method developed by Marcano et al. with some modifications [41]. Briefly, 200 mg graphite flakes (Fisher Scientific, Bridgewater, NJ, USA) were dispersed by sonication in 9:1 mixture of H_2_SO_4_ and H_3_PO_4_ (Sigma Aldrich, Saint Louis, MO, USA). After complete dispersion, the mixture was vigorously stirred, whereas 1.2 g of KMnO_4_ (Sigma Aldrich, Saint Louis, MO, USA) was gradually added. The temperature was set to 50 °C, and the mixture was then aged under continuous stirring for 36 h. Then, the solution was slowly poured out into 400 mL of high purity deionized water (HPDW) with 30% H_2_O_2_ (3 mL). The solution under stirring was then heated until boiling (solution turned brown at these conditions) and it was filtered with a 0.8 μm Nylon filter (Fisher Scientific, Bridgewater, NJ, USA). The filtrate was centrifuged (4000 rpm for 4 h), and the supernatant was decanted away. The remaining solid material was then washed in succession with 200 mL of water, 200 mL of 30% HCl (Sigma Aldrich, Saint Louis, MO, USA), and 200 mL of ethanol (Sigma Aldrich, Saint Louis, MO, USA); afterwards, it was again filtered and centrifuged three more times. The material remaining after this extended, multiple-wash process was dispersed in abundant HPDW, and filtered with a 0.45 μm polycarbonate filter. The filtered material was finally freeze-dried.

### 2.2. Synthesis of rGO/ZnS:Mn

An aqueous solution (80 mL) of the as-synthetized GOx at 0.2 mg/mL was probe-sonicated for 20 min to further increase the solution dispersion and reduce the particles’ agglomeration. Then, a 10 mL solution of both ZnSO_4_ at 2 M and MnSO_4_ at 0.1 M was slowly pipetted to the GOx solution under vigorous stirring. Afterwards, 10 mL of 2 M Na_2_S solution was gradually added. The mixture was stirred at room temperature (RT), and then aged for 14 h at 70 °C under controlled reflux. To further favor the GOx reduction, the solution was thermally treated in a Teflon-lined autoclave and maintained at 170 °C for 5 h. The flocculate was removed from the supernatant by ultracentrifugation, and copiously rinsed with HPDW, and then freeze-dried. The final products were re-dispersed in HPDW producing a deep orange solution when exposed to Ultraviolet (UV) light, which is a clear indicator of the formation of ZnS:Mn QDs, and were then employed for further ex-situ characterization.

### 2.3. Functionalization of rGO/ZnS:Mn with Folic Acid

The functionalization was performed by mixing rGO/ZnS:Mn (0.5 mg/mL) with 10 mM of 1-Ethyl-3-(3-dimethylaminopropyl)carbodiimide (EDC, Sigma Aldrich, Saint Louis, MO, USA) and 20 mM of N-Hydroxysuccinimide (NHS, Sigma Aldrich, Saint Louis, MO, USA) in 0.1 M MES and 0.5 M NaCl at pH 6.3 for 1h at RT. Then folic acid (0.3 mg/mL) was added to the mixture under vigorous stirring for 24 h. The excess FA was removed using repeated washing with nanopure water by ultracentrifugation (Mw. cut-off 50 kDa).

### 2.4. Characterization

The optical absorption spectra were recorded using a UV-Vis spectrophotometer (DU 800, Beckman Coulter, Brea, CA, USA). A Cary Eclipse spectrophotometer was employed to collect the emission spectra in phosphorescence mode. The emission spectra were acquired from 400 to 700 nm, using slit widths of 10 and 20 nm for emission and excitation, respectively. A Molecular Devices microplate reader was used for absorption, fluorescence and luminescence cellular assays. The phase and crystalline structures of the composite were analyzed using an X-Ray diffractometer (XRD), Rigaku Smart-Lab Japan, equipped with a Cu Kα radiation source, and operated at an accelerating potential of 40 kV and a tube current of 44 mA. The FT-infrared (IR) spectra were recorded using a Mode Tensor 27 (Bruker, Billerica, MA, USA) in the range of 100–4000 cm^−1^. The surface morphology, size and degree of particle abundance were studied using sing a JSM-7500F (JEOL, Tokyo, Japan) field-emission scanning electron microscope (FE-SEM) and a JEM-2200FS (JEOL, Tokyo, Japan) Cs-corrected high-resolution transmission electron microscope (HRTEM) operated at 200 kV. The hydrodynamic size and zeta potential of the nanocomposite (before and after conjugation with FA) were determined by dynamic light scattering (DLS) using a Malvern Zetasizer Nanoseries (Malvern Instruments, Malvern, UK). To do this, the samples were dispersed in phosphate-buffered saline (PBS) solution at pH = 7.4, and in Eagle’s minimum essential medium (DMEM) supplied with 10% fetal bovine serum (FBS).

### 2.5. Doxorubicin Loading and Release Studies

DOX (0.5 mM) was loaded onto rGO/ZnS:Mn and FA-rGO/ZnS:Mn by a stirring incubation process in PBS buffer (pH 8.0) overnight at RT as reported by Ma et al. [42]. To remove the excess of DOX, the solution was further ultracentrifuged at 14,800 rpm for 10 min. The precipitate was then re-suspended and stored at 4 °C. The drug loading content and entrapment efficiency were determined via:

(1) $$\mathrm{Loading}\;\mathrm{Factor}\;\left(\text{\%}\right)=\frac{\mathrm{weight}\;\mathrm{of}\;\mathrm{absorbed}\;\mathrm{drug}}{\mathrm{weight}\;\mathrm{of}\;\mathrm{nanoparticles}}$$

(2) $$\mathrm{Entrapment}\;\mathrm{Efficiency}\;\left(\text{\%}\right)=\frac{\mathrm{weight}\;\mathrm{of}\;\mathrm{absorbed}\;\mathrm{drug}}{\mathrm{weight}\;\mathrm{of}\;\mathrm{added}\;\mathrm{drug}}$$

The release studies were performed at three pH values (5.3, 7.4 and 8.0) as previously reported [26,42,43]. Briefly, DOX-FA-rGO/ZnS:Mn was dispersed into PBS buffer (2 mL) at a specific pH value and at 37 °C in absence of light. The solutions were centrifuged at 10,000 rpm at different times and the supernatant was then taken and its absorbance was measured at 480 nm. The concentrations of the released drug were calculated by the Beer–Lambert Law.

### 2.6. Cell Culture

Human breast adenocarcinoma cells, MDA-MB 231 were grown in DMEM media, supplemented with 10% fetal bovine serum, 100 units/mL penicillin and 100 μg/mL streptomycin, HEPES (1%) and cultured in a 5% CO_2_ humidified atmosphere at 37 °C. Similarly, National Institute of Health (NIH)-3T3 cells were grown in DMEM media, supplemented with 10% fetal bovine serum, 100 units/mL penicillin and 100 μg/mL streptomycin and cultured in a 5% CO_2_ humidified atmosphere at 37 °C.

### 2.7. Cell Cytotoxicity Assay

The cytotoxicity effects of rGO/ZnS:Mn, DOX-rGO/ZnS:Mn, FA-rGO/ZnS:Mn, DOX-FA-rGO/ZnS:Mn and free DOX were performed following our previous protocol [9]. Briefly, MDA-MB-231 and NIH-3T3 cells were seeded in 96-well plates and grown until about 80% of confluence. The nanocomposite doses without DOX ranged from 7.5 to 250 μg/mL. The DOX loaded nanocomposite doses tested ranged from 0.5 to 6 μg/mL. The cells were incubated at 37 °C for 24 h. To investigate the effect of the dispersion method used for the stock-solution preparation on the viability of the MDA-MB-231 cells, rGO/ZnS:Mn and FA-rGO/ZnS:Mn were incubated with MDA-MB-231 cells at different doses (7.5 to 250 μg/mL). To study the effect of the cellular uptake time on the viability of the MDA-MB-231 cells, 50 μg/mL of rGO/ZnS:Mn QDs, FA-rGO/ZnS:Mn QDs, DOX-FA-rGO/ZnS:Mn were incubated with MDA-MB-231 cells for different times (24, 48 and 72 h).

### 2.8. Lactate Dehydrogenase Assay

Membrane integrity of cells exposed to rGO/ZnS:Mn, DOX-rGO/ZnS:Mn, FA-rGO/ZnS:Mn, DOX-FA-rGO/ZnS:Mn and free DOX was evaluated by lactate dehydrogenase assay (LDH). MDA and NIH cells were seeded in 96-well plates and grown until about 80% of confluence. Subsequently, the media were removed, and then 100 μL of complete medium supplemented with the composites at different concentrations (ranging from 7.5 to 250 μg/mL) was added, and three wells with only fresh complete medium were used as a positive control. Cell-free supernatants from the cultures were collected, and centrifuged at 2000 rpm for 5 min; 50 μL of the media supernatant was then added to a fresh 96-well plate along with LDH assay reagent (Promega, Madison, WI, USA). After incubating for 40 min, the absorbance values were recorded at 490 nm with SpectraMax M5/M5e (Molecular Devices, Sunnyvale, CA, USA). Positive control was obtained by treating control cells with 10 μL of lysis solution for 45 min before centrifugation, which was used for calculating the percentage of LDH release, i.e., activity of released LDH in a treated sample over the activity of maximal LDH released in the control.

### 2.9. FA-Mediated Drug Release and Cellular Uptake

Cellular uptake by MDA (folate positive receptor) and NIH (folate negative receptor) cells were evaluated using confocal laser scanning microscopy. MDA cells were seeded in 96-well plates with complete medium, and incubated overnight. The cells were then treated with FA-rGO/ZnS:Mn, DOX-FA-rGO/ZnS:Mn, DOX-rGO/ZnS:Mn and DOX for 6 h. Afterwards, the cells were washed with PBS to remove unbound nanoparticles, and fixed with formaldehyde. In the case of the cellular uptake of FA-rGO/ZnS:Mn by MDA cells, the cells were imaged using Nikon Eclipse Ti Series Inverted Microscope (Nikon, New York, NY, USA). In the case of drug release studies, the cells were stained with Hoechst (Thermo Scientific, Chicago, IL, USA), and images were collected with a BD Pathway 855 High-Content Bioimager (BD Biosciences, San Jose, CA, USA) with a 20× NA075 objective (Olympus, Tokyo, Japan) and coupled to a laser auto-focus (2 × 2 binning) with flat field correction using standard excitation, emission, and dichroic filters blue (Hoechst/CellMask Blue) and red (Ethidium Homodimer 1, DOX) channels. Image analysis was performed using the custom MATLAB^®^ (The Mathworks, Natick, MA, USA) software as previously described [44,45].

### 2.10. Statistical Analysis

One-way analysis of variance (ANOVA) was carried out to evaluate the significance of the experimental data vs. the control, and Student’s *t*-test statistical analyses was used to compare two groups together (e.g., rGO/ZnS:Mn vs. FA-rGO/ZnS:Mn). *p* value < 0.05 was chosen as the significance level, *p* values indicated with (#) correspond to *p* < 0.05 vs. control, and (*) for *p* < 0.05 between two different groups.

## 3. Results and Discussion

### 3.1. Characterization of rGO/ZnS:Mn

**Scheme 1:**
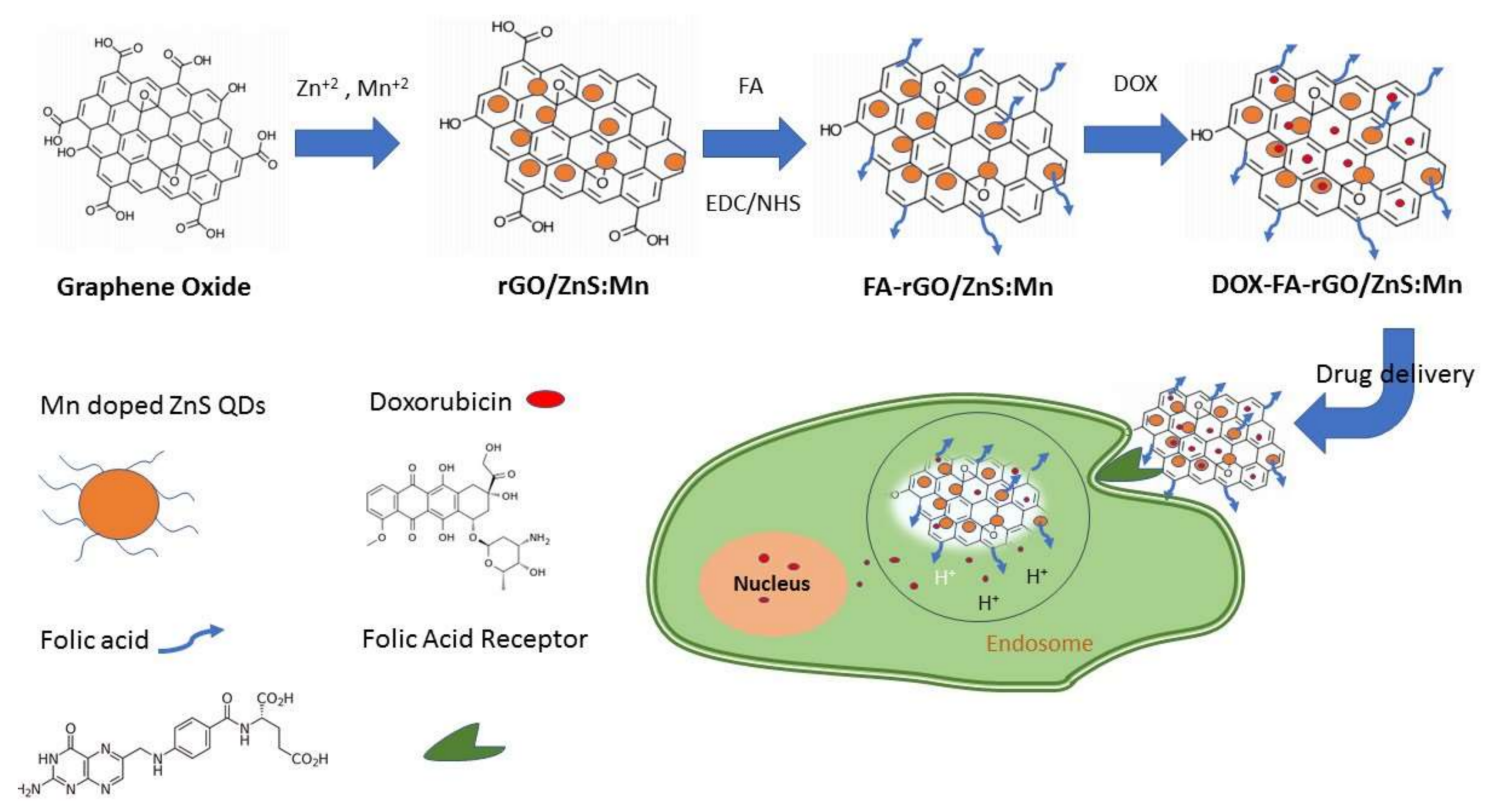
Illustration of the preparation approach of FA-rGO/ZnS:Mn as a drug delivery system to release DOX into the cancer cell nucleus (drawn not to scale).

**Figure 1:**
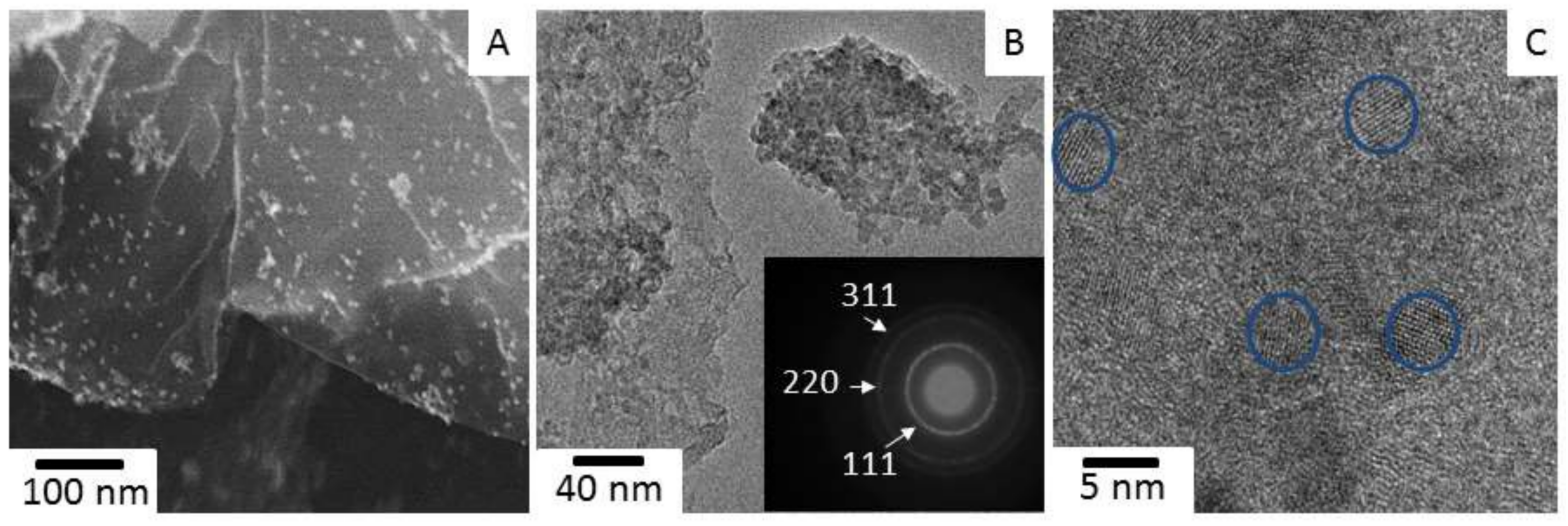
(**A**) SEM image; and (**B**,**C**) HRTEM of rGO/ZnS:Mn nanocomposite. Inset in (**B**) shows the SAED rings taken on the area of Figure 1B.

The preparation approach and subsequent internalization of FA-rGO/ZnS:Mn into the targeted cells are shown in Scheme 1. GOx is first obtained following the improved Hummers’ method and was further dispersed in HPDW. GOx is then decorated with ZnS:Mn QDs following a well-studied hydrothermal-assisted co-precipitation method used in similar graphene/QDs systems [46,47,48]. The surface morphology, particle size and particle abundance of the so-synthesized nanocomposite are depicted in Figure 1. SEM image analysis indicates that the samples consist of particles in the nanoscale that are well dispersed onto the surface of graphene flakes (see Figure 1A). Closer looks (TEM images in Figure 1B,C) reveal graphene flakes with sizes around 200–500 nm. On the surface there can be seen nanoparticles are near-spherical and homogeneously distributed with a high degree of crystallinity, whose sizes range from 3 to 5 nm. To determine the phase of the nanoparticles, we performed selective area electron diffraction (SAED) studies on the region shown in Figure 1B. The SAED pattern shows three major diffraction rings (see inset of Figure 1B), which are indexed to the diffraction planes of the cubic sphalerite phase of polycrystalline ZnS [(111), (220) and (311)] [10].

**Figure 2:**
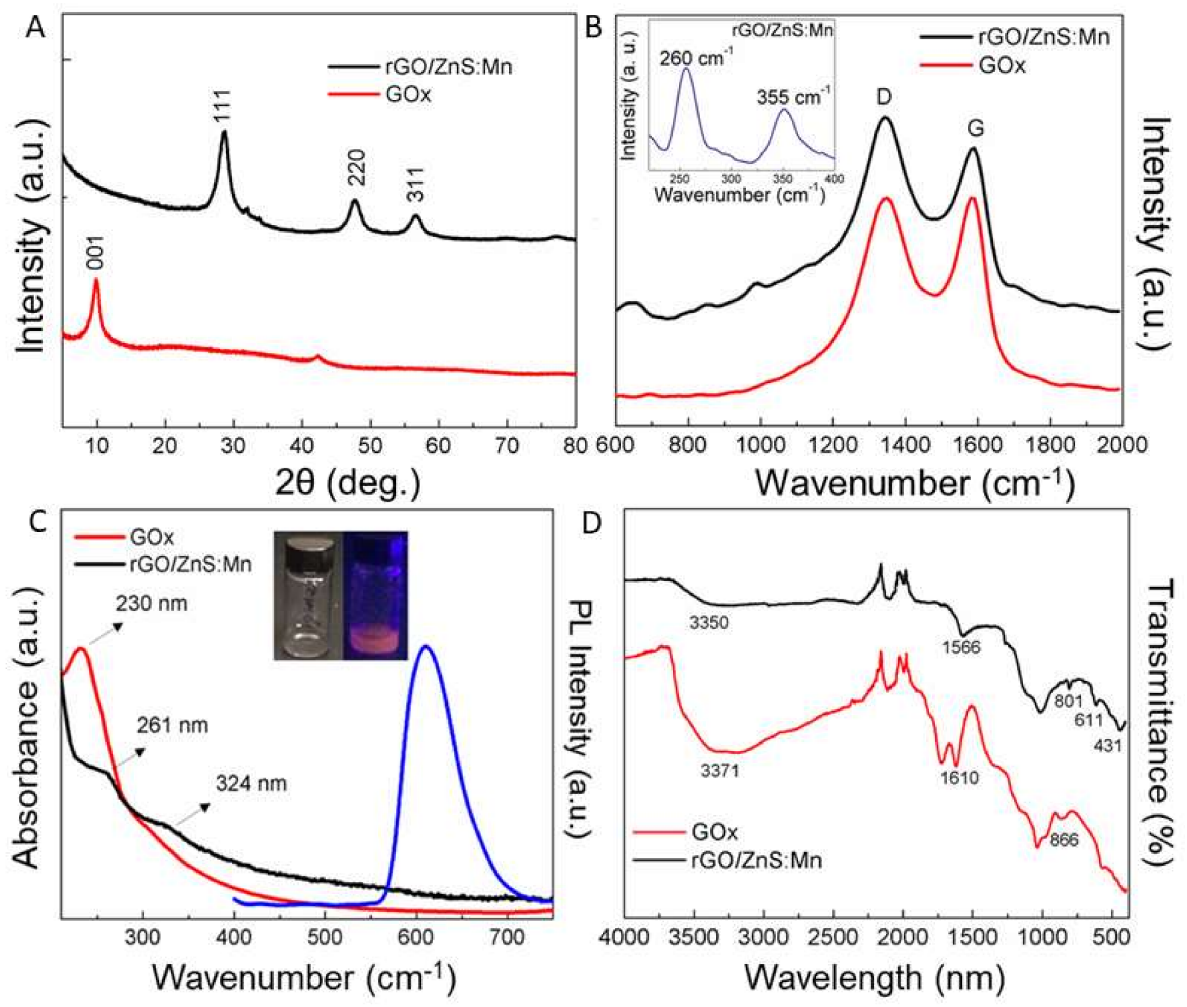
(**A**) XRD patterns; and (**B**) Raman; (**C**) UV/PL; and (**D**) FT-IR spectra of GOx and FA-rGO/ZnS:Mn. Inset in (**B**) shows the low-frequency Raman spectrum of ZnS:Mn. Inset in (**C**) shows the optical images of FA-rGO/ZnS:Mn in absence and presence of UV light (365 nm).

Such findings were further confirmed by X-ray diffraction carried out on bulk powders of rGO/ZnS:Mn and GOx, as shown in Figure 2A. This Figure shows three prominent broad peaks at 2Ɵ values of 28.5, 47.5 and 57.6°, which are consistent with both the preferential cubic crystallization of ZnS:Mn QDs [49], and the SAED analysis. The average crystallite size was calculated to be ~2.1 nm by means of Scherrer’s formula, which is in agreement with the electron microscopy analysis.

Figure 2A also shows a diffraction peak at 2Ɵ = 10° ascribed to the [0002] reflection of GOx. The appearance of this peak in rGO/ZnS:Mn is associated to our robust thermal reduction process [50]. To further corroborate the reduction of GOx, we conducted Raman spectroscopy on rGO/ZnS:Mn and GOx, and the generated spectra are depicted in Figure 2B. We observed a shift of the D and G bands to lower-frequency regions, which is typically attributed to GOx reduction. A marked increase on the D/G intensity ratio in the nanocomposite’s spectrum (1.30 vs. 0.99) was also noted, which resulted from a disorder on graphene structure and possibly from structure deformation due to the QDs decoration. A less-intense vibration band peaked at 345 cm^−1^ was also observed (see inset of Figure 2B), which corresponds to the longitudinal optical modes of cubic ZnS [51,52]. The optical properties of the nanocomposite were also studied, and the spectra are displayed in Figure 2C. The absorbance spectra show an absorption peak centered at 230 nm associated to π–π* transitions of aromatic C=C in GOx, which is red-shifted (~31 cm^−1^) when ZnS:Mn is coupled. This shift is indicative of the restoration of the π-conjugation network of graphene as a result of the hydrothermal reduction process [36,53]. An additional peak centered at 324 nm is attributed to the exciton transition of ZnS [18]. The emission spectrum of rGO/ZnS:Mn also depicted in Figure 2C shows a prominent orange emission band at ~598 nm, which is related to the internal Mn^2+^ ion transition between the ^4^*T*_1_ first excited state (spin 3/2) and the ^6^*A*_1_ ground state (spin 5/2) of ZnS:Mn [18]. Such emission is also displayed in the inset of Figure 2C, where optical images of rGO/ZnS:Mn dispersions were taken in absence and presence of 325 nm light. The functional group density on the surface of GO and rGO/ZnS:Mn was studied using FTIR spectroscopy. As depicted in Figure 2D, the intensity of the characteristic vibrations of GOx at 3371 cm^−1^ (v_str._ O-H), 1720 cm^−1^ (v_str._ C=O), 1610 cm^−1^ (v_bend_ aromatic C=C, O–H), 1230 and 1050 cm^−1^ (v_str._ Epoxy C–O and alkoxy C–O) was significantly reduced in rGO-ZnS:Mn, which is indicative of the reduction of GOx. Some additional vibrations centered at ~670 cm^−1^ and 1100 cm^−1^ were also observed, which correspond to the stretching vibration modes of Mn-S and Zn-S, respectively [54]. Taken together, these characterization techniques furnish clear evidence of the successful decoration of ZnS:Mn QDs onto the surface of GOx (which was reduced via a hydrothermal treatment), and reveal the efficiency of our synthetic approach to obtain a multifunctional nanocomposite.

### 3.2. Functionalization of rGO/ZnS:Mn Nanocomposite

**Figure 3:**
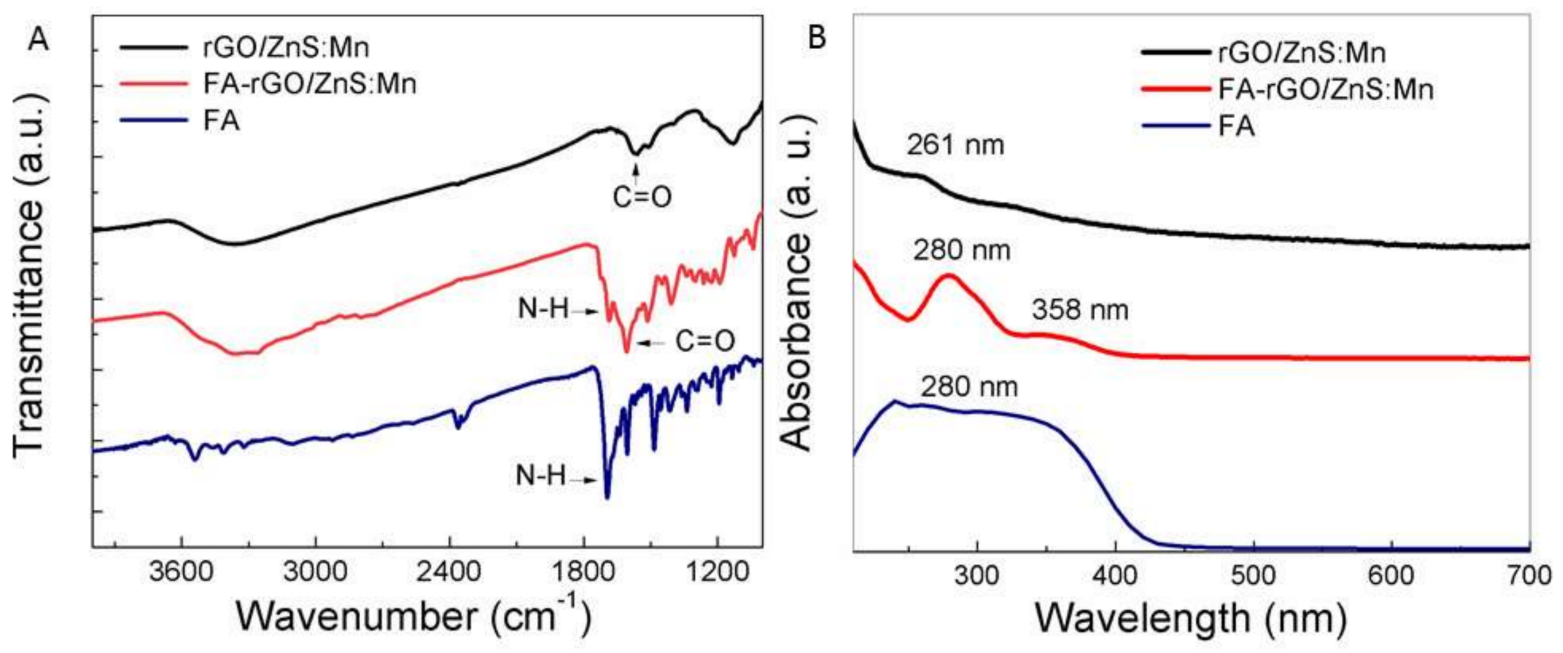
(**A**) FT-IR; and (**B**) UV-Vis spectra of rGO-ZnS:Mn, FA-rGO-ZnS:Mn and free folic acid (FA) showing the FA conjugation via carbodiimide covalent crosslinking approach.

To target cancer cells that exhibit folate receptors, a carbodiimide covalent crosslinking approach to functionalize the nanocomposite with folic acid (FA) was used. To confirm the conjugation, FT-IR studies on rGO/ZnS:Mn, FA-rGO/ZnS:Mn and FA were performed (as shown in Figure 3A). The FT-IR spectrum of FA-rGO/ZnS:Mn shows two vibrational bands. One band centered at ~1500 cm^−1^ attributed to the C=O stretching vibration (amide I mode), which was also observed in rGO/ZnS:Mn, and an additional vibrational band peaked at ~1600 cm^−1^ that corresponds to the N–H bending and C–N stretching vibrations (amide II mode), which is typically observed in FA. Both bands evidence the amide formation. To further confirm the conjugation, UV-Vis studies were carried out on rGO/ZnS:Mn, FA-rGO/ZnS:Mn and FA, as shown in Figure 3B. The spectrum of FA-rGO/ZnS:Mn shows the typical absorption peaks of rGO/ZnS:Mn (as described in previous section), and an extra absorption peak centered at ~280 nm, which is distinctive in FA absorption. Notice this peak does not appear in the spectrum of rGO/ZnS:Mn (cf. Figure 2C), as expected. Moreover, the PL spectrum of FA-rGO/ZnS:Mn (see Figure S1) shows a marked quenching of the orange emission band, which indicates the coordination of the carboxyl groups of FA with the Zn^2+^ surface atoms of QDs [20,23].

It is well-documented that conjugation of materials in dispersion leads to increase in the surface charge [30]. By conducting zeta potential tests (see Figure S2A), we determined that FA conjugation of rGO-ZnS:Mn in PBS leads indeed to a charge increase from 5.53 mV to −17.6 mV, which is ascribed to the carboxyl groups of FA that make the nanocomposite more stable in suspension. Extended studies reveal that adding serum (e.g., supplemental FBS) to cellular media such as DMEM does change the surface charge of the nanocomposite when compared to those obtained using non-supplemented cellular media. Wei and co-workers [30] reported similar findings for GOx and rGO samples, which (despite showing different surface charges in PBS media) exhibit similar zeta potential values when dispersed in media supplemented with FBS [30]. This phenomenon was adjudicated to the absorption of proteins on GOx and rGO surfaces. In our study, the protein–graphene interaction was found to improve the dispersion stability of the nanocomposite. Hydrodynamic sizes of the nanocomposite (as shown in Figure S2B) remained stable for a period of 24 h, when dispersed in cellular media supplemented with FBS. This was not the case for the dispersion in absence of FBS (see Figure S2B). Our findings are compatible with recent studies reported, which indicate that proteins play a critical role in stabilizing nanostructures (including graphene) upon the formation of protein corona on the surface (further favored by FA functionalization) that in turn generates extra electrostatic repulsion avoiding re-agglomeration [30,55,56]. This finding is of paramount importance for drug loading and in-vitro cellular studies.

### 3.3. DOX Loading on FA-rGO/ZnS:Mn and Its Subsequent Release

**Figure 4:**
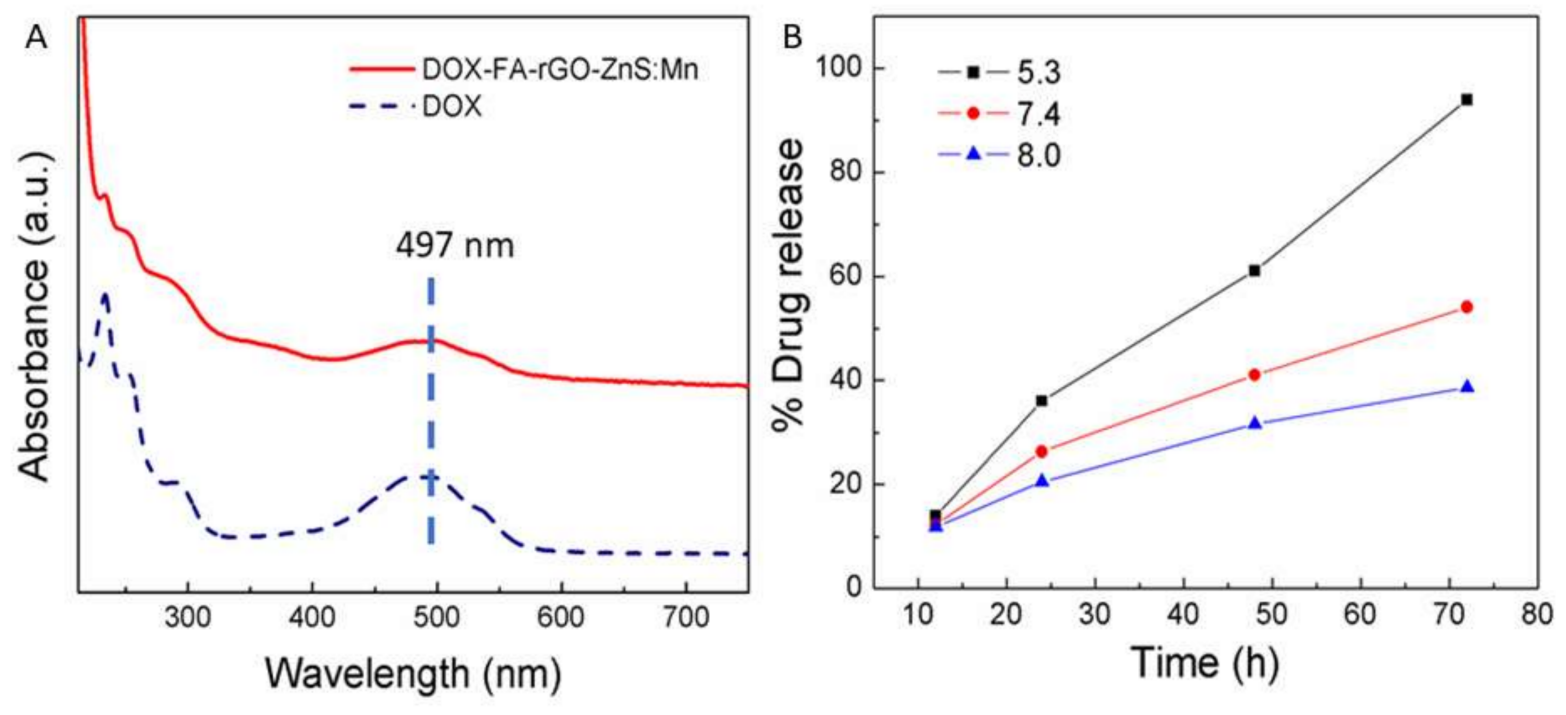
(**A**) UV-Vis absorbance spectra of free DOX and DOX-FA-rGO/ZnS:Mn nanocomposite; (**B**) Cumulative percent drug release over time at different pH conditions.

Fluorescent FA-rGO/ZnS:Mn nanocomposite was loaded with DOX, which was selected as the model anticancer drug. The drug loading was studied by UV-Vis spectroscopy. The recorded spectra of DOX-FA-rGO/ZnS:Mn and free DOX are shown in Figure 4A. The spectrum of DOX-loaded nanocomposite exhibits the typical absorption peaks of FA-rGO/ZnS:Mn (as described in previous section), and an additional absorption peak centered at ~497 nm, which corresponds to the absorption peak of DOX. This evidences that DOX is adsorbed on the graphene surface, which is driven by π–π stacking and hydrophobic interaction. Notice that this peak does not appear in the spectrum of FA-rGO/ZnS:Mn (cf. Figure 3B). It should be pointed out that the enhanced loading is likely due to the chelation of DOX and Zn^2+^ atoms located on the surface of the nanocomposite, as reported by Cai and co-workers who found that this type of interaction improves the loading efficiency of DOX in ZnO-based nanocomposite [57]. To quantify our results, the weight values of added and absorbed DOX and ZnS:Mn were inserted in Equations (1) and (2), obtaining an estimated loading efficiency of ~34% with an entrapment efficiency of ~60%. This value was higher than that reported for GO-PEG/ZnS:Mn nanocomposite that have a loading factor of 10% [28]. Our drug delivery system unlike this system contains reduced GO, which has a higher population of aromatic ring structures as a result of reduction, and is more hydrophobic, both factors enhanced the DOX loading. This finding was also reported by Kim and co-workers who observed that the drug loading capacity of PEG-BPEI-rGO nanocomposite are remarkably higher than its unreduced counterpart (PEG-BPEI-GO with a loading factor of 10%) [58]. Another point that should be noted is the fact that the loading efficiency for DOX-loaded non-functionalized nanocomposite is ~30% higher than that of its functionalized counterparts, indicating that FA relatively hinders the DOX loading. This is attributed to the bulkiness of FA, which disrupts the interactions of graphene and DOX, thus reducing the efficiency of DOX loading. In contrast, the non-functionalized nanocomposite is less dispersed, suffers re-agglomeration, and is unsuitable for targeting folate-rich cancer cells.

Since the release of DOX is sensitive to the acidity of media [35], we measured the drug release at three different pH values (5.3, 7.4 and 8.0) in PBS buffer and at 37 °C over time. The results depicted in Figure 4B reveal that the release of DOX from the drug delivery nano-assembly is pH dependent, being more favored under acidic conditions (pH 5.3) reaching a release of up to 95% after 70 h. This is due to the fact that under acidic conditions DOX is protonated, which affects its interactions with both graphene and Zn^2+^ and Mn^2+^ ions and in turn promoting its release [17,57]. On the contrary, under alkaline conditions (pH 8.0) the release is almost negligible, even after 48 h the amount of DOX release was merely ~30% while at pH 5.3 is ~65%. This pH discrimination endows our drug delivery nanosystem with extra efficiency to deliver DOX into folate positive cancer cells without potentially inducing toxic response to non-overexpressed cells, which lack folate receptors on their surface and have a more neutral pH [37,59].

### 3.4. Cytotoxicity Assay

**Figure 5:**
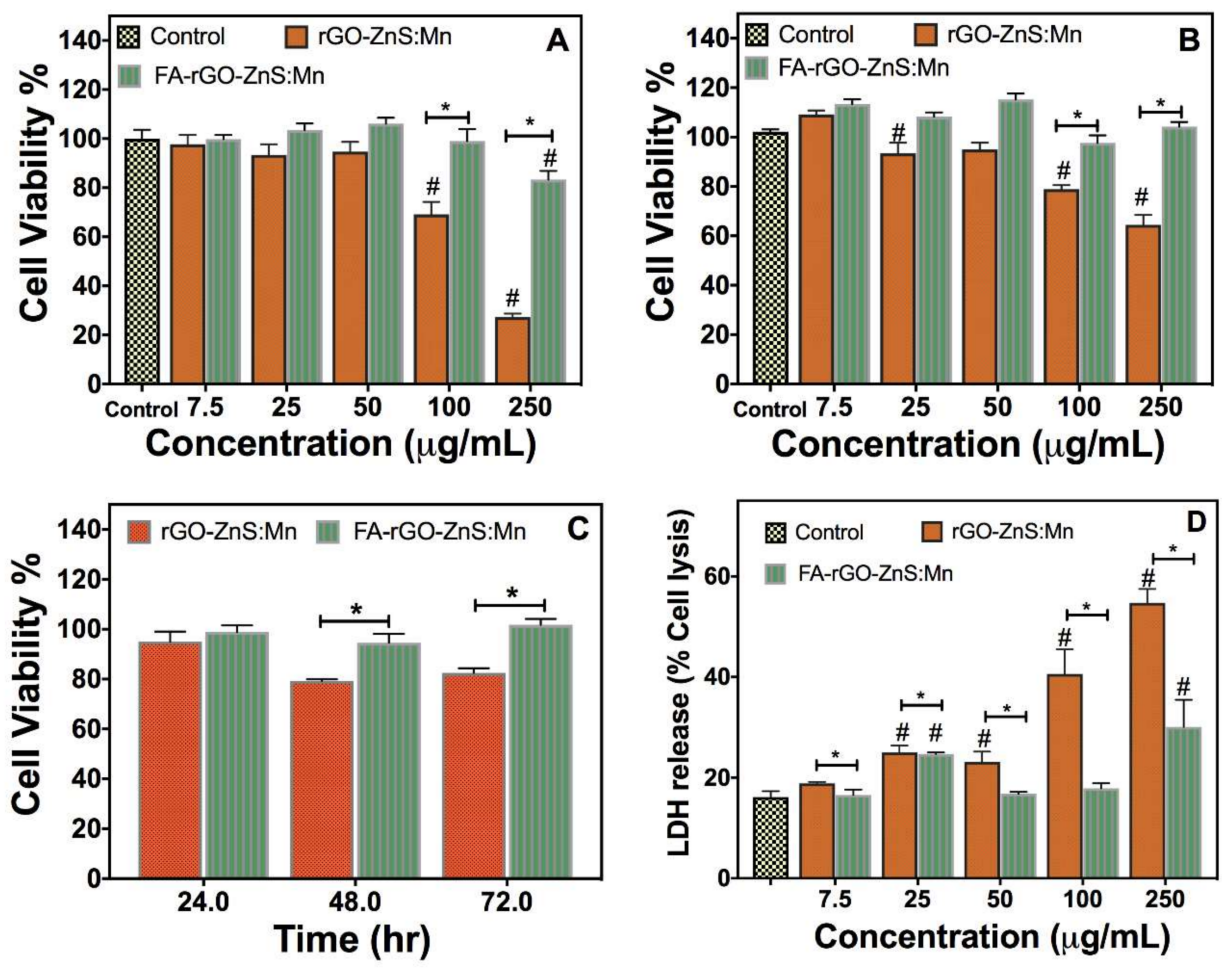
In-vitro MTS cytotoxicity assays showing the (**A**) MDA-MB 231 and (**B**) NIH-3T3 cell viability when exposed to FA-rGO/ZnS:Mn and rGO/ZnS:Mn after 24 h of incubation; (**C**) The incubation time dependence on MDA-MB 231 cell viability after an exposure dose of 50 µg/mL for FA-rGO/ZnS:Mn and rGO/ZnS:Mn; (**D**) LDH release of MDA-MB 231 cells at different exposure doses of FA-rGO/ZnS:Mn and rGO/ZnS:Mn for 24 h. (**D**) Data are presented as the mean ± standard deviation (SD; *n* = 3). *p* value < 0.05 was chosen as the significance level, *p* values indicated with (#) correspond to *p* < 0.05 vs. control, and (*) for *p* < 0.05 between two different groups.

The cytotoxic response of our drug delivery nanosystems was evaluated using two different cell lines: The breast cancer cell line MDA-MB 231 (FA receptor positive) and NIH-3T3 cell line (FA receptor negative). Both cells were exposed to FA-rGO/ZnS:Mn and rGO/ZnS:Mn at a dose range of 7.5–250 µg/mL, and then their viability at 24 h was assessed via MTS assays, which are depicted in Figure 5A,B. No statistically significant reduction in the viability of both cells was observed for FA-rGO/ZnS:Mn through the whole range of doses. In the case of rGO/ZnS:Mn, a detectable decrease in the viability of both cells was observed at doses greater than 50 µg/mL (^#^ *p* < 0.05 vs. control). Note that at 250 µg/mL the MDA-MB 231 cell viability was reduced up to a 70%, a more pronounced reduction than the observed with NIH-3T3 cells. A similar result was also reported by Chowdhury et al., who observed that NIH3T3 cells were more resistant than Hela cells to graphene nanoribbons [60]. Nevertheless, in both cells, it was clear that FA functionalization reduce significantly the toxicity of the composite. Several factors could be ascribed to this effect that include the passivation of rGO/ZnS:Mn after functionalization, which has been reported to be an effective pathway to increase the biocompatibility of other graphene based systems, such as PEG-rGO [61], Dextran-GO [62] and BSA-GO [63]. Surface passivation decreases the strong hydrophobic interaction between the membrane wall of the cells and the edges/corners of graphene flakes, which could induce several toxic responses [64]. Recent reports have also indicated that the cell viability is dramatically altered in presence of graphene-based nanocomposites at longer exposures. We have thus studied the effect of time exposure on the viability of MDA-MB231 cells at the maximum particle dose (50 µg/mL), where no toxic effect was detected for rGO/ZnS:Mn at 24 h. The MTS assays at 24, 48 and 72 h are shown in Figure 5C. As expected, a statistically significant decrease on the cell viability exposed to rGO/ZnS:Mn after 24 h was observed. No cell viability reduction was observed for FA-rGO/ZnS:Mn even after 72 h of exposure. This clearly indicates that the FA functionalization plays a pivotal role in the biocompatibility of graphene oxide/ZnS:Mn QDs. However, its net hydrophobicity could cause several disruptions in the cellular membrane. To shed light upon this issue, we assessed the membrane integrity by LDH release in MDA-MB231 cells exposed to different doses (7.5–250 µg/mL) of rGO/ZnS:Mn and FA-rGO/ZnS:Mn. The LDH assays at 24 h are depicted in Figure 5D. An abrupt increase (up to ~60%) in the LDH release was observed at doses above 50 µg/mL in rGO/ZnS:Mn. No substantial difference on the LDH release caused by FA-rGO/ZnS:Mn (when compared to the control) was also noticed even up to 100 µg/mL. These results further confirm that FA functionalization passivates the surface of rGO-ZnS:Mn rendering it as a potential inhibitor of cellular membrane damage. To discard whether the sonication method used to disperse the graphene/QD nanocomposite is affecting our results, we dispersed the nanocomposite via ultrasonication (compared to bath sonication used in this study) and studied its effect on cell viability and LDH release in MDA-MB-231 cells. As shown in Figure S3, a similar trend was observed which indicates that the FA surface passivation is the main predictor of toxic response in rGO/ZnS:Mn regardless of the dispersion method used. It should be mentioned that supplying high acoustic energy (e.g., above 500 J/mL) to the dispersions can cause ZnS:Mn to be detached from graphene.

### 3.5. Chemotherapeutic Effect

**Figure 6:**
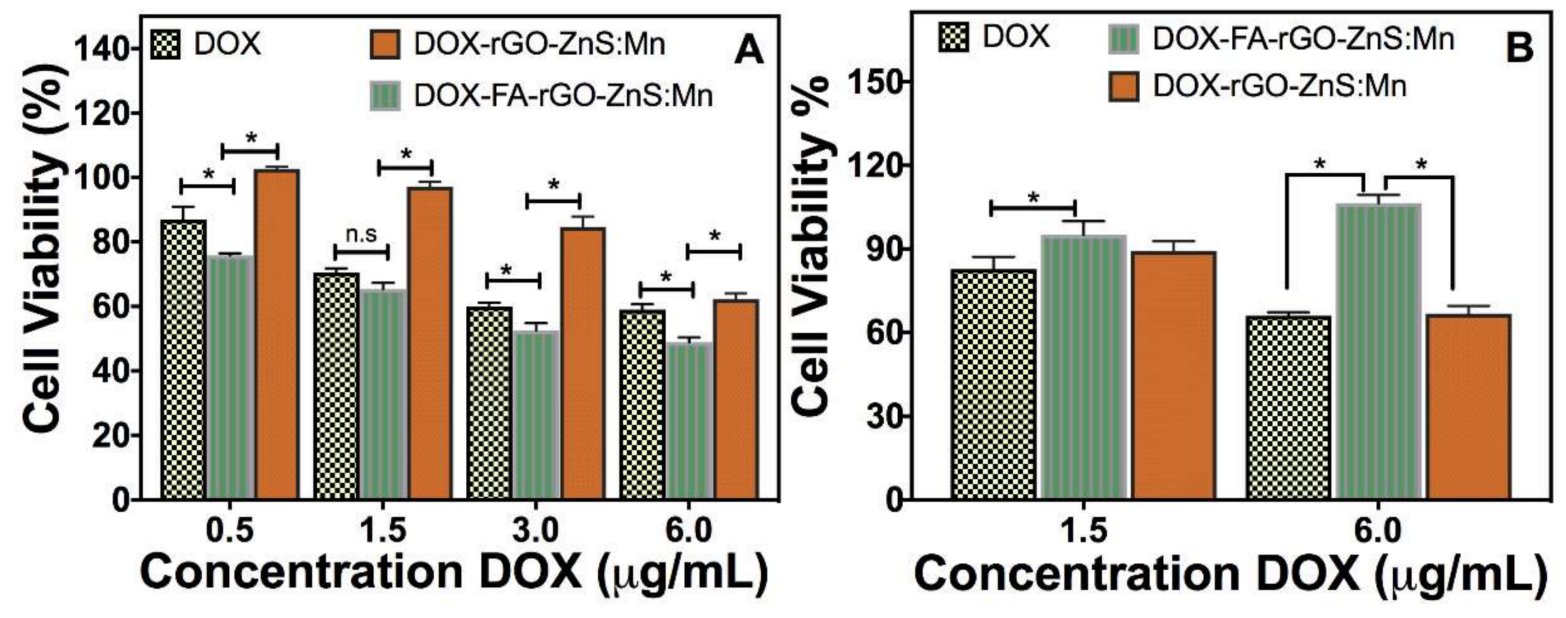
Chemotherapeutic effect of DOX-FA-rGO/ZnS:Mn and DOX-rGO/ZnS:Mn in (**A**) MDA-MB-231 cancer cells and (**B**) NIH-3T3 cells after 24 h of incubation. *p*-values are shown as follows: ns, non-significant and * *p* < 0.05 is considered as significant.

As previously discussed, the addition of FA on the surface of rGO/ZnS:Mn minimizes its cytotoxicity. Our next assessment was to test whether FA functionalization could also enhance the drug delivery efficiency and specificity of rGO/ZnS:Mn. DOX lacks discrimination among normal and cancerous cells which leads to acute side effects, such as cardiomyocytes degradation [65]. In this regard, FA-rGO/ZnS:Mn offers the possibility to transport DOX to targeted cells without affecting neighboring tissues. The FA-crosslinked approach could be an excellent strategy to target cancer cells that overexpress folate receptors on their surfaces for graphene-based drug delivery systems. Following these reported findings, we exposed MDA-MB 231 cancer cells that exhibit high overexpression of folate receptors to FA-rGO/ZnS:Mn and rGO/ZnS:Mn at different DOX doses ranging from 0.5 to 6 µg/mL for 24 h. This is depicted in Figure 6A, where free DOX is also added for comparison purposes. It was observed that DOX-FA-rGO/ZnS:Mn shows a more pronounced chemotherapeutic effect than free DOX and DOX-rGO/ZnS:Mn for all the range of doses, with differences in cell viability reductions (statistically significant) of up to ~7% and ~21%, respectively. It is also noted that the cell viability was further reduced down to 50% at 3 µg/mL of DOX-FA-rGO/ZnS:Mn exposure. This killing efficiency is not as high as some other graphene-based nanosystems reported in the literature, nevertheless the DOX concentrations tested here are below the concentrations frequently used (>10 µg/mL) in such reports [66,67]. Our findings are well consistent with the drug release studies (cf. Figure 4B), which indicate that approximately 40% of the drug loaded is released under acidic conditions at 24 h. We have thus at this dose extended the exposure time up to 48 h and noticed a more marked decrease in the cell viability close to 20%, which was closer to free DOX (data not shown). It is worthy of remark that unlike other reports our drug loaded non-functionalized system did not cause any significant toxicity in the DOX dose range of 0.5–3 µg/mL, further evidencing the contribution of FA functionalization in improving the efficiency of graphene/QDs-based drug delivery systems.

The pending question was to evaluate whether FA functionalization can improve the selectivity of our drug delivery system to discriminate positive folate receptor cancer cells. We have selected NIH-3T3 cells, which do not overexpress folate receptors to address this question. We exposed the NIH-3T3 cells to two DOX concentrations (1.5 and 6 µg/mL) and assessed its cell viability after an exposure of 24 h. As depicted in Figure 6B, no statistically significant decrease (i.e., no chemotherapeutic effect) in cell viability was observed in DOX-FA-rGO/ZnS:Mn for both doses. We also observed that at 6 µg/mL both free DOX and DOX-rGO/ZnS:Mn show a marked cell viability reduction of up to ~60%. This clearly indicates that FA functionalization endows rGO/ZnS:Mn with an effective capability to discriminate positive folate receptor cancer cells enhancing its drug loading and release, and efficiency as a compact drug delivery nanocomposite [53].

### 3.6. Fluorescent Imaging and Cellular Uptake

**Figure 7:**
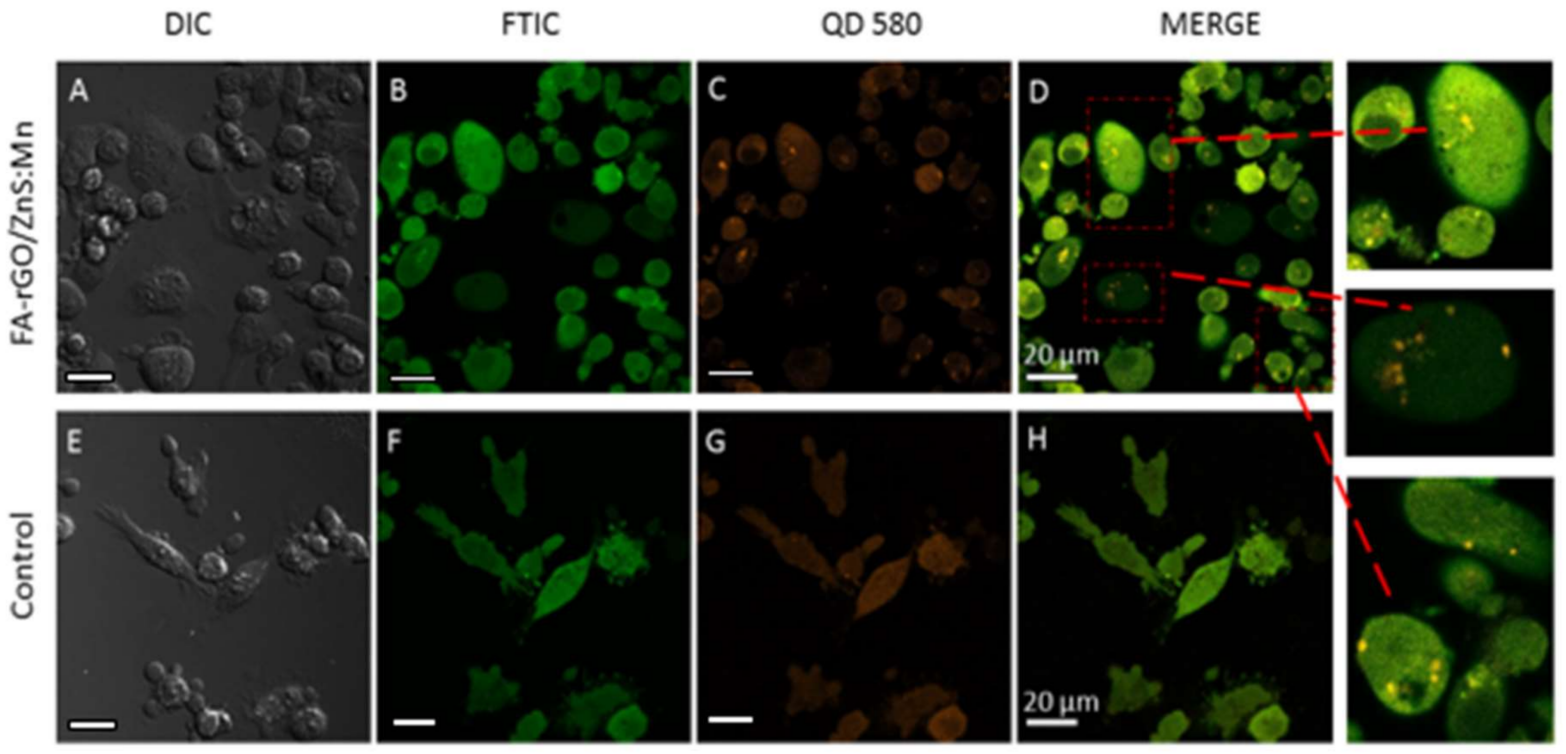
Bright-field (DIC), fluorescence (FTIC and QD 580) and merged microscopic images of MDA-MB 231 cells in the presence (**A**–**D**) and absence (**E**–**H**) of 100 µg/mL FA-rGO/ZnS:Mn after incubation for 6 h. Scale bar = 20 µm.

The cellular internalization and intracellular drug release behavior of the nanocomposite were assessed by confocal microscopy. MDA-MB 231 cells modified with GFP were incubated with FA-rGO/ZnS:Mn for 6 h, and after copious washes, the cells were fixed for imaging, as depicted in Figure 7A–D. For comparison purposes, no treated cells (control) were imaged under the same imaging conditions (see Figure 7E–H). The images were recorded using the FTIC and QD 580 emission channels that correspond to the cells intrinsic emission (due to its modification with GFP), and orange emission of FA-rGO/ZnS:Mn, respectively. As shown in merged Figure 7D, we observed that some intense orange signals are randomly distributed in the cells, which could be ascribed to the characteristic fluorescent emission of folic acid functionalized rGO/ZnS:Mn, whose presumably given the size and surface charge of the nanocomposite are taken up by the cells via folate-mediated endocytosis [68]. Note that the control sample showed minimal autofluorescence. Closer looks (see insets of Figure 7D) show that FA-rGO/ZnS:Mn labeled the surfaces and cytoplasm of the cells with orange fluorescence in both the GD 580 channel and merged image in the spectral range of 580–630 nm.

**Figure 8:**
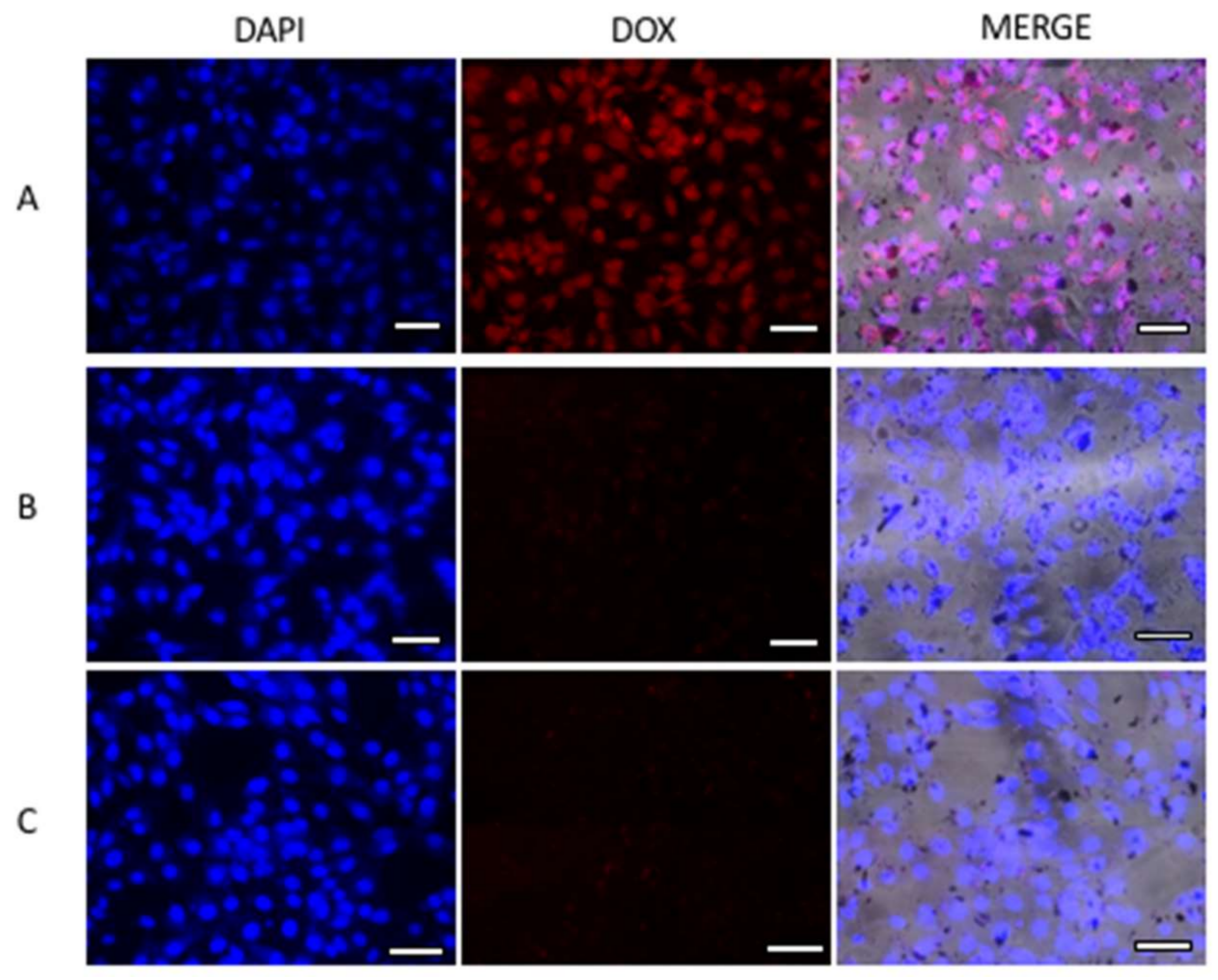
CLSM optical images of MDA-MB 231 cancer cells incubated for 6 h with: (**A**) DOX-FA-rGO/ZnS:Mn; and (**B**) DOX-rGO-ZnS:Mn; (**C**) CLSM optical images of NIH-3T3 cells incubated with DOX-FA-rGO/ZnS:Mn for 6 h. Scale bar = 20 µm.

We further assessed the intracellular drug release of DOX into the nucleus by tracking the red fluorescence of DOX. MDA-MB 231 cells were incubated with DOX-FA-rGO/ZnS:Mn and DOX-rGO/ZnS:Mn at DOX 1.5 µg/mL, from which a detectable chemotherapeutic effect was tested (cf. Figure 6A). Additionally, NIH-3T3 cells that do not overexpress folate receptors were used as negative control, and incubated with DOX-FA-rGO/ZnS:Mn. After incubation for 6 h, the cells were fixed and the nuclei stained with HOESTCH to verify the DOX release into the cells, and then the fluorescence of DOX and cell nuclei stain were compared. The CLSM optical images are collected and depicted in Figure 8. As shown in Figure 8A, DOX-FA-rGO/ZnS:Mn displays a high-intense red fluorescence of DOX in the nucleus, which evidences its high specificity and ability to internalize and effectively deliver DOX inside the nucleus through a folate mediated endocytosis. In contrast, DOX-rGO/ZnS:Mn showed weaker red fluorescence signals (see Figure 8B), which could be ascribed to the absence of FA functionalization. This is compatible with the cell viability assay analysis. A similar optical pattern was observed in Figure 8C that corresponds to the fluorescence images of NIH-3T3 cells, indicating that the functionalization with FA renders DOX-rGO/ZnS:Mn high specific to target preferentially positive folate receptor cells (MDA-MB 231). Moreover, FA functionalization eases dispersion stability, enhances drug loading/release and is a potential inhibitor of toxic response in graphene/quantum dot systems.

## 4. Conclusions

We have successfully developed a new drug-delivery nanocomposite composed of reduced graphene oxide decorated with ZnS:Mn quantum dots. We have shown that the FA functionalization inhibits the toxic response of the nanocomposite when exposed to breast cancer cells even for 72 h. Specifically, surface passivation with folic acid decreases the strong hydrophobic interaction between the membrane wall of the cells and the edges and corners of graphene flakes, which reportedly induces several toxic responses. FA functionalization also enables the nanocomposite to be well-dispersed and stable in cell culture media (regardless of the dispersion method used) without detectable re-agglomeration, which is suitable for enhancing the loading and release of DOX into folate-rich cancer cells. The killing efficiency of FA-rGO/ZnS:Mn was 50% at DOX doses of 3 µg/mL, which is far below the doses frequently reported (>10 µg/mL) in the literature. FA functionalization also improves the selectivity of this drug delivery platform for discriminating positive folate receptor cancer cells. FA-rGO/ZnS:Mn is thus an excellent candidate as theranostic agent to treat breast cancer. This piece of research represents a step forward to improve media-dispersability, drug loading/release, tracking, and toxicity of graphene/quantum dots drug-delivery systems.

## Supplementary Materials

The following are available online at http://www.mdpi.com/2079-4991/8/7/484/s1.
