# Supplementary material for: Graphene Oxide/ZnS:Mn Nanocomposite Functionalized with Folic Acid as a Nontoxic and Effective Theranostic Platform for Breast Cancer Treatment
Source: Nanomaterials (Basel). 2018 Jun 30;8(7):484. doi: 10.3390/nano8070484 (PMC6071040; doi:10.3390/nano8070484)
Supplement: Supplementary file 1 [file nanomaterials-08-00484-s001.pdf]

Graphene Oxide/ZnS:Mn Nanocomposite Functionalized with Folic Acid as a Nontoxic and Effective Theranostic Platform for Breast Cancer Treatment

Daysi Diaz-Diestra [1,2,*], Bibek Thapa [1,4], Dayra Badillo-Diaz [5], Juan Beltran-Huarac [3,*], Gerardo Morell [1,4] and Brad R. Weiner [1,2]

[1] Molecular Sciences Research Center, University of Puerto Rico, San Juan, PR 00926, USA; bibech.thapa@gmail.com (B.T.); gerardo.morell@upr.edu (G.M.); bradrweiner@gmail.com (B.R.W.)
[2] Department of Chemistry, University of Puerto Rico, San Juan, PR 00925-2537, USA
[3] Department of Environmental Health, Center for Nanotechnology and Nanotoxicology, Harvard T. H. Chan School of Public Health, Boston, MA 02115, USA
[4] Department of Physics, University of Puerto Rico, San Juan, PR 00925-2537, USA
[5] Department of Biology, University of Puerto Rico, San Juan, PR 00925-2537, USA; dnbd1118@hotmail.com
* Correspondence: daysi.diaz@upr.edu (D.D.-D.); juanbh@email.unc.edu (J.B.-H.)

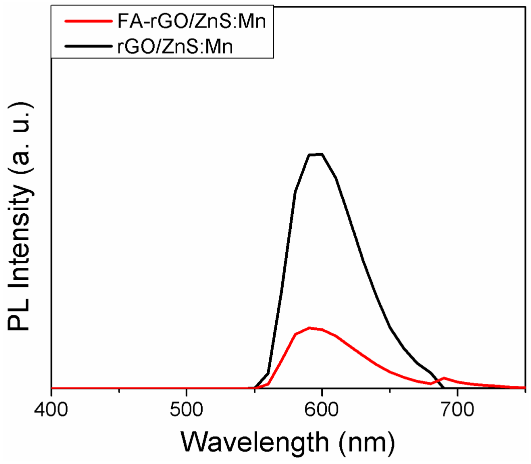

Figure S1. PL spectra of FA-rGO/ZnS:Mn and rGO/ZnS:Mn under 325 nm light excitation.

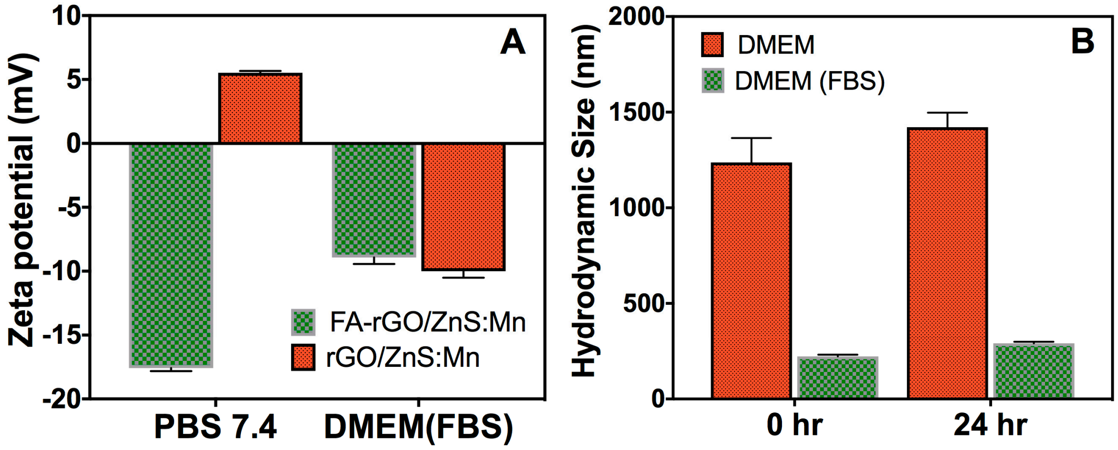

Figure S2. (A) Zeta potential of FA-rGO/ZnS:Mn and rGO/ZnS:Mn in PBS (pH 7.4) and cellular media supplemented with FBS. (B) Mean hydrodynamic size of FA-rGO/ZnS:Mn dispersed in both DMEN and DMEM supplemented with FBS at 0 and 24 hrs.

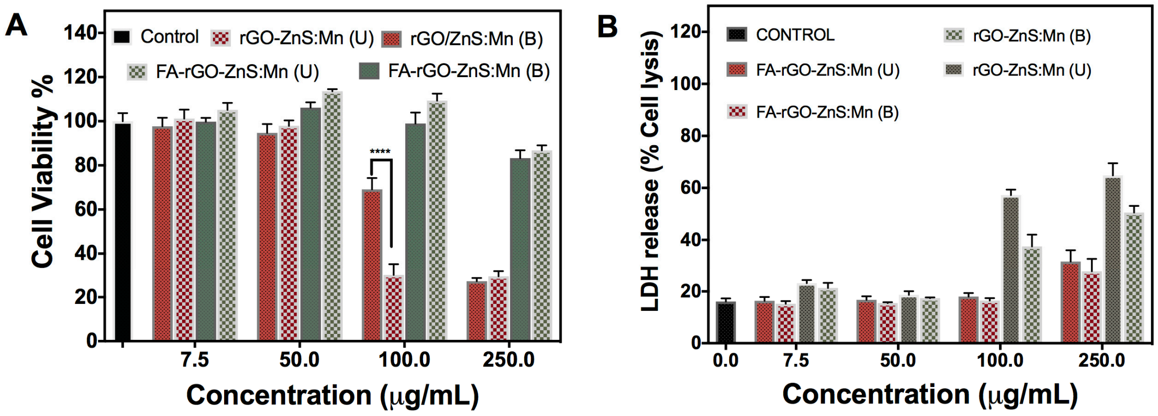

Figure S3. (A) *In-vitro* MTS cell viability and (B) LDH release of MDA-MB 231 cells when exposed to FA-rGO/ZnS:Mn and rGO/ZnS:Mn (which are dispersed through ultrasonication-U and bath sonication-B) at different doses for 24 hrs of incubation.
